# ^1^H-NMR Determination of Organic Compounds in Municipal Wastewaters and the Receiving Surface Waters in Eastern Cape Province of South Africa

**DOI:** 10.3390/molecules25030713

**Published:** 2020-02-07

**Authors:** Adebayo I. Farounbi, Paul K. Mensah, Emmanuel O. Olawode, Nosiphiwe P. Ngqwala

**Affiliations:** 1 Environmental Health and Biotechnology Research Group, Division of Pharmaceutical Chemistry, Faculty of Pharmacy, P.O. Box 94, Rhodes University, Grahamstown 6140, South Africa; kunlefaroline@yahoo.com (A.I.F.); eolawode@yahoo.com (E.O.O.); 2 Institute for Water Research, Rhodes University, Grahamstown 6140, South Africa; kojomens2@hotmail.com

**Keywords:** freshwater, wastewater, metabolites, spectroscopy, nuclear magnetic resonance, chemical shifts

## Abstract

Surface water is the recipient of pollutants from various sources, including improperly treated wastewater. Comprehensive knowledge of the composition of water is necessary to make it reusable in water-scarce environments. In this work, proton nuclear magnetic resonance (^1^H-NMR) was combined with multivariate analysis to study the metabolites in four rivers and four wastewater treatment plants releasing treated effluents into the rivers. ^1^H-NMR chemical shifts of the extracts in CDCl were acquired with Bruker 400. Chemical shifts of ^1^H-NMR in chlorinated alkanes, amino compounds and fluorinated hydrocarbons were common to samples of wastewater and lower reaches or the rivers. ^1^H-NMR chemical shifts of carbonyl compounds and alkyl phosphates were restricted to wastewater samples. Chemical shifts of phenolic compounds were associated with treated effluent samples. This study showed that the sources of these metabolites in the rivers were not only from improperly treated effluents but also from runoffs. Multivariate analyses showed that some of the freshwater samples were not of better quality than wastewater and treated effluents. Observations show the need for constant monitoring of rivers and effluent for the safety of the aquatic environment.

## 1. Introduction

Over the years, freshwater bodies had been the recipient of the wastes generated as a result of human activities and thus have been abused [1,2]. If the wastes were released into the air or soil, they would eventually enter into water bodies as a result of rain and erosions. These result in water pollution, which has become a global problem [3]. Human activities and population increase, coupled with an increase in industrial and farming activities, have led to the generation of more wastes. The United Nations Organization (UNO) sustainable development goals (SDGs) article 6 is to “ensure availability and sustainable management of water and sanitation for all” [4]. Unfortunately, water quality around the world has been in danger and threatening human health, food security and biodiversity [5].

Before the invention of NMR, structural elucidation of a molecule used to take days and months. The discovery of chemical shifts as a result of the variation in NMR frequencies facilitates the process of chemical structural elucidation [6]. In the early days, NMR started with a continuous wave, a system whereby the oscillator frequency was constant while the magnetic field change gradually, and signal amplitude measured as a function of frequency [7]. The early NMR has a weak magnetic field; measurements depended on the energy absorbed. Later, the continuous wave was replaced by pulsed Fourier transform, which involved the application of short, intense radiofrequency pulse over the entire bandwidth of frequencies in which the nuclei resonate. That method allowed all the nuclei falling within the region to be excited simultaneously [8]. Total scan in Fourier transform is independent of the sweep width. The relaxation that occurs immediately after the excitation process is measured as exponentially decaying waves (FID), which are converted to NMR spectra by Fourier transformation [9].

NMR spectroscopy has become an evolving analytical tool in organic and inorganic chemistry and a versatile tool in the analysis and structural determination of bio-macromolecules [10]. NMR spectroscopy is a useful tool in molecular biology, providing a reliable method for atomic resolution and structure determination of biological macromolecules in aqueous solutions similar to natural physiological environments that have posed a challenge to X-rays [11]. It has also proven to be the most powerful technique for quantifying the conformational properties of bio-macromolecules, giving useful information in the rate of enzymatic conversion of substrates to products [12]. The understanding of molecular motion is necessary because enzymes change their conformation several times in the course of catalyzing reactions and these changes commensurate with the rate constants that define the reaction mechanism. NMR spectroscopy is the most powerful tool for determining the residual structures of proteins, whether in folded conformation, intermediates or unfolded disordered proteins [13]. NMR is also a powerful tool for determining the chemical properties of functional groups in bio-macromolecules, such as the ionization states of some groups at enzymes active sites [14]. It provides a unique molecular movement and interaction profiles with information on protein functions, which are necessary for drug development [15]. NMR spectroscopy is a useful tool in drug screening, identification and determination of metabolites interactions with enzymes, receptors and other proteins [16]. The high sensitivity of NMR to protein binding has made it possible for the screening of ligands bindings [15,17].

The ecosystem is a dynamic structure where physical, chemical and biological processes interact, understanding these components is necessary to adequately address the effects of climate change, urbanization, industrial and agricultural activities that are affecting the system [18]. NMR spectroscopy has become a versatile tool in the study of chemical structures and interactions in the soil, water and air. Solid-state NMR is a useful tool in the analysis of soil, especially chemical composition, moisture and organic matter contents [19], and in the determination of soil microbial products and constituents [18]. NMR has been a tool in the study of soil humification processes, aggregate structure, stability, fertility and in the prediction of the response of soil carbon pool to land-use change, agriculture and climate change [20,21].

NMR is a tool to monitor qualitative and quantitative changes of metabolites in the aquatic ecosystem and to examine the presence of external inputs such as contaminants or nutrient enrichment [22,23]. It has been a tool in water quality assessment and monitoring of organisms’ response to pollutants [24]. NMR is useful in monitoring ion exchange in water sediments and nutrient dynamics in the aquatic environment [25]. Navalon et al. [26] analyzed the chemical components of treated wastewater effluents with NMR, and Filho et al. [27] used it to monitor the efficiency of wastewater treatment plants (WWTPs). Information obtained from NMR of wastewater analyses is useful in monitoring, processing and quality control, to ensure that the final effluent released after treatment is fit for public use and to optimize the performance of wastewater treatment plants [28].

In this work, freshwater, wastewater and treated effluent samples were analyzed with ^1^H-NMR spectroscopy to determine the chemical functional groups in the water samples. The objective of this research work is to determine the functional groups of organic compounds in the water samples using ^1^H-NMR spectroscopies as an aid to proper water quality monitoring.

## 2. Results

^1^H-NMR spectroscopy gives better resolution than ^13^C-NMR (i.e., ×4) and more useful in metabolites analysis [29,30], hence ^1^H-NMR is often used in metabolomics of biological fluids containing low concentrations of the metabolites. Standard NMR chemical shifts tables were consulted before arriving at point-by-point analysis shown in this result [31,32,33]. Table 1 shows the functional groups recorded from ^1^H-NMR chemical shifts for freshwater and Table 2 for wastewater samples.

The reference point (0 ppm) is the chemical shifts for protons in tetramethylsilane (TMS). Proton chemical shifts below zero show that more energy is absorbed than the TMS value. ^1^H-NMR chemical shift for dimethylzinc, (CH_3_)_2_Zn, was observed in F2A (Table 1).

The wastewaters and their treated effluents show proton shifts in cyclic pentane and phosphine (1.3–1.4 ppm). Proton shifts in brominated alkanes were only present in Grahamstown treated effluent samples (G2B and G2C) while alkyl iodide was present in Grahamstown and King Williams samples (GS, G1A, G1C, G2A, K1C and K2C).

**Figure 1:**
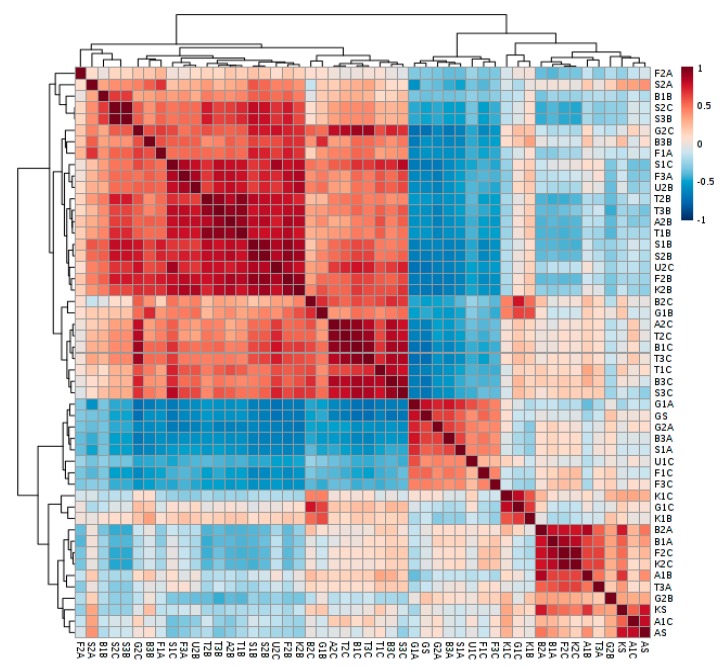
Heatmap showing the correlation of the samples. Positively correlated samples are shown in brown color and negative in blue. Bloukrans River samples: B1A (upstream, autumn); B2A (midstream, autumn); B3A (downstream, autumn); B1B (upstream, winter); B2B (midstream, winter); B3B (downstream, winter); B1C (upstream, spring); B2C (midstream, spring) and B3C (downstream, spring). Buffalo River samples: F1A (upstream, autumn); F3A (downstream, autumn); F1B (upstream, winter); F2B (midstream, winter); F3B (downstream, winter); F1C (upstream, spring); F2C (midstream, spring) and F3C (downstream, spring). Swartkops River samples: S1A (upstream, autumn); S3A (downstream, autumn), S1B (upstream, winter); S2B (midstream, winter); S3B (downstream, winter); S1C (upstream, spring); S2C (midstream, spring) and S3C (downstream, spring). Tyhume River samples: T1A (upstream, autumn); T3A (downstream, autumn); T1B (upstream, winter); T2B (midstream, winter); T3B (downstream, winter); T1C (upstream, spring); T2C (midstream, spring) and T3C (downstream, spring); Grahamstown samples: G1A (wastewater, autumn), G2A (treated effluent, autumn), G1B (wastewater, winter), G2B (treated effluent, winter), G1C (wastewater, spring), G2C (treated effluent, spring), GS (sludge). King Williams Town samples: K1B (wastewater, winter), K2B (treated effluent, winter), K1C (wastewater, spring), K2C (treated effluent, spring). Alice samples: A1B (wastewater, winter), A2B (treated effluent, winter), A1C (wastewater, spring), A2C (treated effluent, spring). Uitenhage samples: U1B (treated effluent, winter), U1C (treated effluent, spring).

^1^H-NMR data obtained from Mnova 14 uploaded to MetaboAnalyst 4.0 were not filtered, and there were no missing values observed. Sample normalization was according to the method reported by Dieterle et al. [34], with the column-wise procedure by sample median. Generalized log transformation (glog 2) was adopted with Pareto data scaling, involving mean-centered divided by the square root of the standard deviation of each variable [34]. Figure 1 shows the correlation heatmap of the samples.

Samples with positively correlated spectral features are shown in brown color and negative in blue. Samples with a correlation coefficient greater than 0.5 are strongly positively correlated while those with −0.5 or lesser values are strongly negatively correlated.

**Figure 2:**
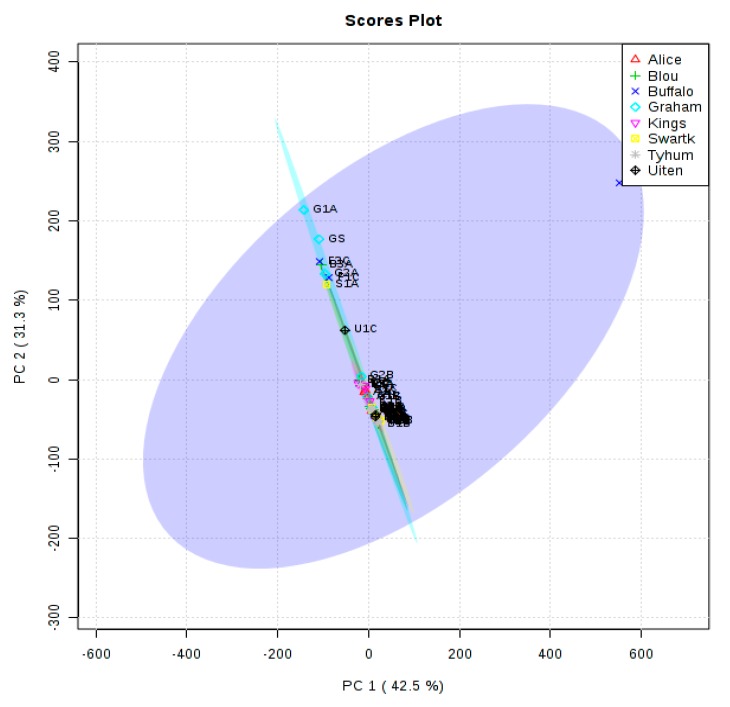
Principal component analysis scores plot between selected principal components (PCs). The variances are in brackets.

The principal component analysis (PCA) was performed using the prcomp package, with the calculation based on singular value decomposition. Figure 2 shows the PCA scores plot for components **1** and **2**.

Two main clusters were identified with some samples not clustered with others. Samples G1A, GS and U1C, appeared not to cluster with other samples. Samples S1A, G2A, F3C, B3A and F1C clustered together, indicating similarities of features. Sample U1C in between the two main clusters might have shared some features with them.

**Figure 3:**
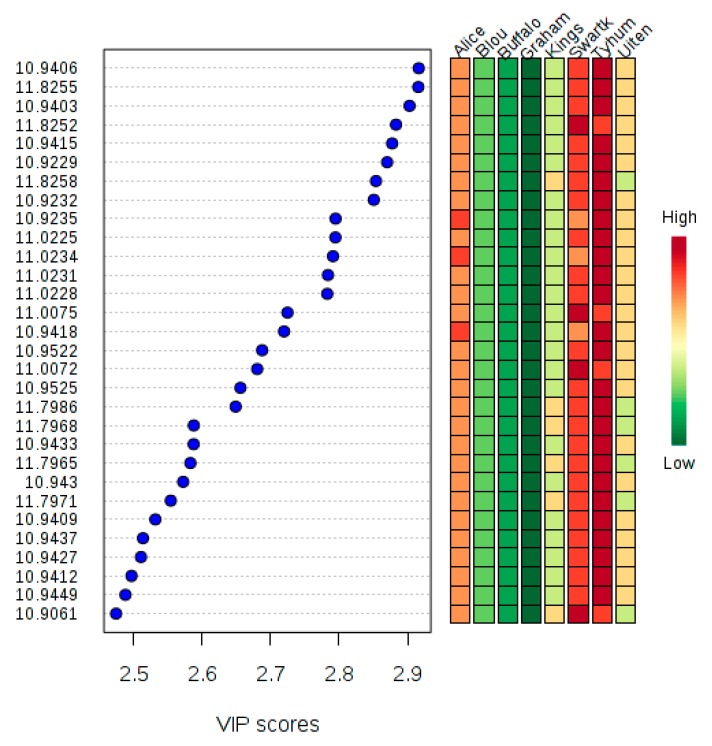
Important features as identified by PLS-DA. The colored box on the right indicates the relative concentrations of the components in the groups listed.

Variable importance in projection (VIP) is a partial least squares-discriminant analysis (PLS-DA) tool for identifying priority features in the samples. These features relate to their abundance in the samples. Figure 3 shows the VIP scores for 30 important features of the samples. However, targeted metabolomics is required to understand the compounds with these features. The Tyhume River had the highest concentrations of the listed features, followed by the Swartkops River.

**Figure 4:**
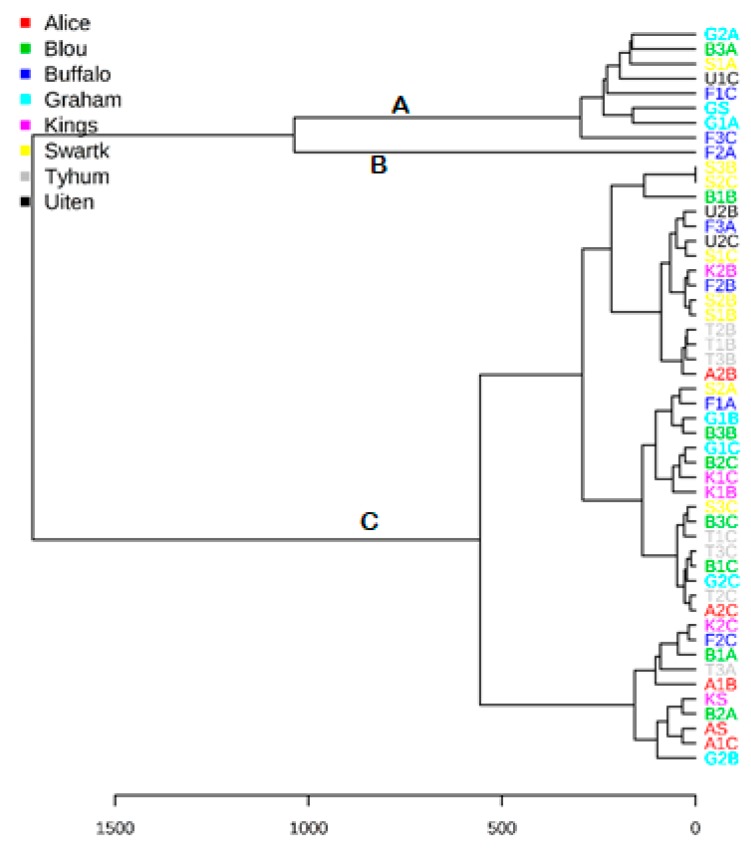
Clustering result shown as a dendrogram. The distance was measured with Euclidean and clustering algorithm with Ward D.

Hierarchical clustering (HC) analysis with the hclust function in package stat organized the samples into homogenous groups with closely related samples grouped [35]. The dendrogram (Figure 4) shows the result of HC analysis, clustering algorithm with Ward’s linkage with similarities between the components measured with Euclidean distance.

Three main clusters A, B and C, were identified with many sub-clusters. Cluster A contained eight samples, while sample F2A was in cluster B, and cluster C had the highest number of samples. Samples from the same geographical location have the same color representation.

## 3. Discussion

Dimethylzinc, observed in Table 1, is a synthetic compound and might have arisen from industrial processes; its presence in the water sample is an indication of industrial pollution [36].

Brominated alkanes are water disinfectants [37], but reports show that some bromides (e.g., brominated trihalomethane) are environmental carcinogens [38]. Alkyl halides are common in industries for the production of refrigerants, propellants, fire retardants and drugs, from where they enter the environment [39]. Proton shifts of amino bonded alkanes were observed in wastewater and treated effluents samples while ^1^H-NMR shifts of triethylamine at 2.5 ppm were restricted to wastewater samples. Alkynes proton shifts (1.9–2.4 ppm) were also present mostly in wastewater and lower reaches of the rivers. ^1^H-NMR shifts of acetonitrile and methacrylonitrile (2–2.05 ppm) were common to all the samples. Acetonitrile is a by-product of methacrylonitrile with various uses as analytical materials in laboratories (e.g., LC-MS), battery production, as solvents in pharmaceuticals and photographic films. Methacrylonitrile is widely used in the preparation of amides, amines and plastics among other uses. Proton shifts similar to HC=CH in furan, imidazole and methenamine were observed in some wastewater and treated effluent samples. Imidazole is an anti-microbial agent present in some drugs, such as analgesics, antifungal, antibacterial and anticancer therapy [40].

The result of multivariate analyses show that the chemical compositions of freshwater might vary for samples with the same origin in the same season. Tyhume samples T1B, T2B and T3B, appeared on the same cluster, but other river samples were not that similar. It also shows that some freshwater samples such as B2A, B3A and B1C were closely related to wastewater, and T1B, T2B and T3B were related to treated effluents. These variations arose from pollutants entering into the rivers at different reaches. The Tyhume River had the highest concentrations of the features listed by PLS-DA VIP, followed by the Swartkops River. These features were of low concentrations in Grahamstown wastewater and Buffalo River. They could serve as the diagnostic compounds for Tyhume River if analyzed with targeted metabolomics.

## 4. Materials and Methods

### 4.1. Study Area

Four major rivers in Eastern Cape Province of South Africa sampled include Bloukrans (upstream 33°19′0.07” S; 26°31′20.9” E; midstream: 33°18′51.4” S, 26°33′11.5” E and downstream: 33°19′07.1” S, 26°34′05.7” E); Tyhume (upstream, 32°36′38.72” S; 26°54′34.15” E; midstream, 32°47′42.95” S, 26°50′88” E and downstream, 32°50′15” S, 26°53′31.27” E), Buffalo (upstream, 32°47′23.74” S, 27°22′10.56” E; midstream, 32°53′49.14” S, 27°23′34.08” E and downstream: 32°56′3.6” S; 27°26′25.18” E) and Swartkops (upstream, 33°42′59.64” S, 25°17′16.43” E; midstream 33°47′31.08” S, 25°24′26.96” E and downstream, 33°47′31.92” S; 25°29′26.26” E). Samples of wastewater and treated effluents were collected from Grahamstown, Alice, King Williams Town and Uitenhage WWTPs. Each river was sampled at three different locations, as indicated.

### 4.2. Materials

All solvents (acetone, chloroform, ethyl acetate and deuterated chloroform CDCl_3_) used were of analytical standards and purchased from Sigma–Aldrich (Johannesburg, South Africa). Anhydrous sodium sulphate purchased from Sigma Aldrich. Rotary evaporator Büchi Rotavapor R-210 with Büchi Heating Bath B-491 was from Büchi Labotechnik, Meierseggstrasse 40, Postfach 9230 Flawil, Switzerland. NMR spectrometer Bruker AvanceTM III HD 400 MHz spectrometer, Topspin 3.5 pls and SampleXPress autosampler were from Bruker BioSpin, Rheinstetten, Germany.

Software materials include MetaboloAnalyst 4.0 designed by McGill University, Parasitology Building, 21,111 Lakeshore Road Ste. Anne de Bellevue, QC, Canada (http://www.metaboanalyst.ca) used for the multivariate analyses. MestReNova 14 software was from Mestrelab Research S.L., Santiago de Compostela, Spain, and used for the analysis of NMR chemical shifts.

### 4.3. Procedure

Sample bottles were prepared by washing in phosphate-free soap, rinsing with deionized water, dry and soaked into acetone for 30 min, rinsed with hexane and dried at 120 °C [41]. The bottles were rinsed three times with sample water at the point of collection. One liter water sample was collected into a prepared bottle at each site. The bottles were tightly covered, labeled and preserved in the icebox to limit the activities of microorganisms in the water samples and later transported to the laboratory for analyses. The organic content of the water samples was extracted within 24 h of collection. The separation of the organic content from the water samples was by the liquid-liquid extraction (LLE) method [42,43]. An exhaustive extraction was achieved by adding 300 mL of organic solvent to an equal volume of water sample (ratio 1:1), and the procedure was repeated three times, alternating chloroform with ethyl acetate. The organic layer was dried over anhydrous sodium sulphate (Na_2_SO_4_), filtered and the solvent removed in vacuo on a rotary evaporator at 35 °C to obtain the extract. Each extracted sample was transferred to a vial and allowed to dry in an oven maintained at 35 °C followed by analysis. About 30 mg of each extract was dissolved in 500 µL of deuterated chloroform (CDCl_3_, 99.9% atom D), transferred into an NMR tube with 5 mm outer diameter and the tubes queued up on Bruker 400 NMR spectrometer for analysis.

^1^H-NMR chemical shifts of the extracts in CDCl_3_ were acquired at 300 K on NMR spectrometer using a PULprog Zg30. For the ^1^H-NMR with the spectra obtained at 400.13 Hz by taking 16 scans without prior dummy scans, spectra width of 20.0254 ppm, receiver gain of 32 with time and frequency domain of 32,767 and 262,144 points, respectively, and acquisition time of 4.096 s. The ^1^H-NMR spectra were processed and analyzed using MestReNova 14. The NMR signals were calibrated with the chemical shift of the residual CDCl_3_ signal at δ values of 7.26 ppm relative to zero value of tetramethylsilane (TMS). The processing of the spectra involved phase correction by global algorithms, full automated baseline correction with Bernstein polynomials at degree 5, smoothing using the Whittaker Smoother method at a normal mode, zero filling along t1 from 32,768 (32k) to 65,536 (64k) and normalized by the largest peak set at a value of 100. The analysis of the spectra was carried out, including positive peak picking with a noise factor of 50 using an interactive default option and parabolic interpolation with a maximum number of peaks of 10000. The spectra were stacked and aligned (to compensate for the intrinsic acidity of the samples).

The ^1^H-NMR spectra of the water samples were separately saved as ASCII text files (*.txt), then imported to Microsoft Excel and saved as CSV comma (*.csv) files. The CSV files were uploaded to the MetaboloAnalyst 4.0 software followed by multivariate analyses. The following general procedures were carried out on the data; checking for missing values, filtering using median intensity value, quartile normalization with Log 2 transformation, Pareto scaling and cross-validation of the normalized dataset by permutation tests using the LOOCV method with the performance measure set at Q^2^.

## 5. Conclusions

^1^H-NMR chemical shifts showed that some compounds such as alkyl halides were not effectively removed from wastewaters since similar shifts appeared in treated effluent samples. Some compounds with proton shifts in wastewater samples such as methyl bromide were not observed in treated effluents, indicating total removal during treatment. Variable importance in projection (VIP) identified some features of priority, most of which were present in the Tyhume River and could be diagnostic of it if taken further with targeted metabolomics. The result shows that NMR is useful in the analysis of the water samples.

## Figures and Tables

**Table 1:** ^1^H-NMR chemical shifts (relative to TMS at δ = 0) of freshwater samples. The shifting proton is in bold.

δ (ppm)	Compound	Sample
−0.4	(CH_3_)_2_Zn	F2A
0.1	Cyclopropane	B2A, B3A, F1A, F2A, S1A, S3B, T1A, T1B, T2B, T3A, T3B
0.8–0.9	(CH_3_)_4_C (CH_3_)_3_CH	B1A, B1B, B1C, B2A, B2B, B2C, B3A, B3C, F1A, F1C, F2A, F2B, F2C, F3A, F3C, S1A, S1B, S1C, S2A, S2B, S2C, S3B, S3C, T1A, T1B, T1C, T2B, T2C, T3A, T3B, T3C
1.0–1.2	CH_3_CH_2_OH (CH_3_CH_2_)_2_CO (CH_3_)_2_COH	B1A, B2C, F1A, F1C, F2C, F3C, S2A, T1C, T2B, T3A, T3C
1.2–1.3	CH_3_CH_2_CH_3_	B1A, B1B, B1C, B2A, B2B, B2C, B3A, B3C, F1C, F2B, F2C, F3A, F3C, S1A, S1B, S1C, S2A, S2B, S2C, S3B, S2C, T1A, T1B, T1C, T2B, T2C, T3A, T3B, T3C
1.3–1.4	CH_2_P(CH_3_)_3_ 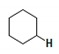	B1B, B1C, B2A, B2B, B2C, B3A, F2B, F2C, F3A, S1B, S1C S2A, S2B, S2C, S3B, T1A, T1B, T2B, T3A, T3B, T3C
1.4–1.5	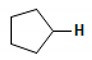	B2B, B2C, B3A, B3C, F2B, F3A, S2A, S2B, S2C, S3B, S3C, T2B, T2C, T3B
1.5–1.69	Chlorinated alkane (CH_3_)_3_C-Cl	B2A, B2B, B2C, B3A, B3C, F1A, F2A, F2B, S2C, S3B, T1B, T2B, T3B, T3C
1.7–1.8	Brominated alkane BrC(CH_3_)_3_; BrCH_2_CH_3_,	B1A, B2A, F1A, F1C, S2C, S3C
1.8–1.9	CH_3_CH_2_I	B1A, B1B, B1C, B2C, B3A, B3C, F2C, F3C, S1C, T3A, T3C
1.9–2	Propyne HC≡C-Me (HC≡C)_2_CH	B1C, B2B, B2C, F1A, F2B, F3A, S1B, S1C, S2A, S2B, S2C, S3B, S3C, T1B, T1C, T2B, T2C, T3B
1–4	RNH_2_ amino	B2A, B2C, F2B, S2B
2–2.05	Acetonitrile, methacrylonitrile CH_3_-C≡N	B1A, B1C, B1B, B2B, B2C, B3C, F1A, F1C, F2A, F2B, F3A, F3C, S1A, S2A, S2B, S2C, S3B, S3C, T1A, T1B, T1C, T2B, T2C, T3A, T3B, T3C
2–2.2	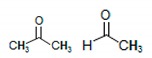 carbonyl compounds	B1A, B1C, B2A, B2C, B3A, B3C, F1A, F1C, F2B, F2C, F3A, S1B, S2B, S1C, S2C, S3B, S3C, T1B, T3B, T3C
2.3–2.4	HC≡CH acetylenic	B1C, B2B, B2C, F1A, F2B, F2C, F3C, F2C, F3C, S2C, T1A, T1C, T2C, T3A
2.4–2.5	(CH_3_CH_2_)_2_CO (CH_3_CH_2_)_3_N	B1C, B3A, F1A, F3C, S1A
2.2–3	Ar–C–H benzylic	F1C, F2B, S1A, S1C, S2B, S2C, S3C, T1C, T3A
2.7–2.8	CH_3_Br bromides	B2C, B3A, F1C, S3C
2.8–2.9	(CH_3_)_2_SO_2_	S3C
3–3.1	(CH_3_)_2_CHCl chlorides	B1A, B2A, F1A, F1C
3.3–4	HC–OH alcohols HC–OR ethers Alkyl halides	B1A, B1B, B1C, B2A, B2C, B3A, B3C, F1A, F2A, F2C, F3A, S1A, S2A, S2B, S2C, T1A, T1C, T2B, T3A, T3B, T3C
3.5	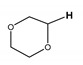 Dioxane	B1C, F1A
3.6–3.7	BrCH_2_CH_2_Br	B1B, B2C, F1A, F2C, F3C, S1A, S2B, S3B, S3C, T1A, T1B, T3A
3.7–4.1	RCOO–CH esters	B1C, B2C, B3A, F1A, F3C, S1A, S1B, S1C, T1A, T2C, T3A
4–4.5	HC–F fluorides	B2A, B2B, B2C, B3A, F1C, F2B, F2C, S1A, S1C, S2C, S3C, T3A, T3C
4.9–5	CH_2_Br_2_	F3A, S3C
4.5–5.2	ArOH phenolic	B1B, B1C, B2A, B2B, B3C, F2A, F2B, F3A, S1B, S2A, S2B, S2C, S3B, S3C, T1A, T1B, T1C, T2B, T2C, T3B, T3C
4.6–5.5	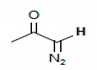 vinylic	B1B, B1C, B2A, B2B, B2C, B3A, B3C, F1A, F2B, F3A, S1A, S1B, S1C, S2A, S2B, S2C, S3B, S3C, T1A, T1B, T1C, T2B, T2C, T3A, T3B, T3C
5.0–5.1	PhCH_2_Cl chlorides	F3A, S2C, B1B, B1C, B2B, B2C, B3C, F1A, F2B, S1B, S1C, S2A, S2B, S3B, T2B, T3B, T3C
6.9–8.5	C=CH shift in heterocyclic compounds	B1B, B1C, B2A, B2C, B3A, B3C, F1A, F1A, F1C, F2A, F2B, F3A, S1B, S1C, S2B, S3B, S3C, T1B, T1C, T2B, T2C, T3A, T3B

Bloukrans River samples: B1A (upstream, autumn); B2A (midstream, autumn); B3A (downstream, autumn); B1B (upstream, winter); B2B (midstream, winter); B3B (downstream, winter); B1C (upstream, spring); B2C (midstream, spring) and B3C (downstream, spring). Buffalo River samples: F1A (upstream, autumn); F2A (midstream, autumn), F3A (downstream, autumn); F1B (upstream, winter); F2B (midstream, winter); F3B (downstream, winter); F1C (upstream, spring); F2C (midstream, spring) and F3C (downstream, spring). Swartkops River samples: S1A (upstream, autumn); S3A (downstream, autumn), S1B (upstream, winter); S2B (midstream, winter); S3B (downstream, winter); S1C (upstream, spring); S2C (midstream, spring) and S3C (downstream, spring). Tyhume River samples: T1A (upstream, autumn); T3A (downstream, autumn); T1B (upstream, winter); T2B (midstream, winter); T3B (downstream, winter); T1C (upstream, spring); T2C (midstream, spring) and T3C (downstream, spring).

**Table 2:** ^1^H-NMR chemical shifts (relative to TMS at δ = 0) of wastewater, treated effluents and sludge samples. The shifting proton is bold.

δ, ppm	Description	Sample
0.1	Cyclopropane	KS, AS, A1C, A2C, G1A, G1B, G2A, K1B, K1C, K2B, K2C
0.8–0.9	(CH_3_)_4_C (CH_3_)_3_C	GS, KS, A1B, A1C, A2B, A2C, G1A, G1B, G1C, G2A, G2B, G2C, K1B, K1C, K2B, K2C, U1C, U2B, U2C
1.0–1.2	CH_3_CH_2_OH (CH_3_CH_2_)_2_CO (CH_3_)_2_COH	GS, KS, AS, A1C, G1A, G1C, G2A, G2B, G2C, K1B, K1C, K2C, U1C
1.2–1.3	CH_3_CH_2_CH_3_	GS, KS, A1B, A1C, A2B, A2C, G1A, G1B, G1C, G2A, G2B, G2C, K1B, K1C, K2B, K2C, U1C, U2B, U2C
1.3–1.4	CH_2_P(CH_3_)_3_ 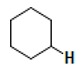	AS, KS, A1B, A1C, A2B, A2C, G1A, G1B, G1C, G2A, G2B, G2C, K1B, K1C, K2B, U1C, U2B, U2C
1.4–1.5	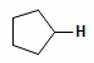	AS, A1B, A1C, A2C, G2B, G2C, K1B, K1C, K2B, U1C, U2B, U2C
1.5–1.69	Chlorinated alkane (CH_3_)_3_C-Cl	GS, KS, A1B, A2B, A2C, G1A, G1B, G1C, G2A, G2B, G2C, K1B, K1C, K2B, U1C, U2B, U2C
1.7–1.8	Brominated alkane BrC(CH_3_)_3_; BrCH_2_CH_3_,	G2B, G2C
1.8–1.9	CH_3_CH_2_I	GS, G1A, G1C, G2A, K1C, K2C
1.9–2	Propyne HC≡C-Me (HC≡C)_2_CH	GS, KS, A2B, A2C, G1A, G1B, G1C, G2A, G2B, G2C, K1B, K1C, K2B, U1C, U2C
1–4	RNH_2_ Amine	KS, GS, A1B, A1C, G1A, G1B, G1C, G2B, G2C, U1C
2–2.05	Acetonitrile, methacrylonitrile CH_3_-C≡N	GS, KS, AS, A1B, A1C, A2B, A2C, G1A, G1B, G1C, G2A, G2C, K1C, K2B, U1C, U2B, U2C
2–2.2	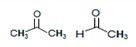 carbonyl compounds	GS, AS, AIB, A2B, A2C, G1A, G1B, G1C, K1B, K1C, K2B, K2C, U1C, U2B
2.3–2.4	HC≡CH acetylenic	A1C, G1C, G2A, K2C
2.4–2.5	(CH_3_CH_2_)_2_CO (CH_3_CH_2_)_3_N	GS, AS, A1B, A1C, A2C, G1B, G2A, G2B, K1B, K1C, K2B, K2C, U1C, U2C
2.2–3	Ar–C–H benzylic	GS, KS, A1C, A2B, G1B, G2A, G2B, G2C
2.7–2.8	CH_3_Br	GS, G1A, G1C, K1B, K1C
2.9–3	HC≡C-Ph	K2C
3.3–3.5	PhCOC≡CH	U2B
3.6–3.7	BrCH_2_CH_2_Br	KS, G1A, G1C, G2A, G2C, K1C, K2B, K2C
3.4–4	CH_2_: Alkyl halides, Alcohols, Ethers	GS, KS, A1B, A1C, A2B, A2C, G1A, G1B, G2A, G2B, K1C, K2B, K2C, U1C, U2B, U2C
4–4.1	MeCOOCH_2_CH_3_ (CH_3_)_2_CHCl	GS, KS, A2C, G1A, G1B, G2A, G2B, K2C, U1C
4–4.5	RCH_2_OH	GS, KS, AS, A2C, G1B, G2A, G2B, G2C, K1B, U1C, U2C
4.5–5	PhCH_2_Cl CH_2_Br PhCH=CH_2_	A1B, A2B, A2C, G1B, G2C, K1B, K2B, U2B, U2C
5.0–5.1	PhCH_2_Cl	A1B, A2B, A2C, G1B
5.3–5.5	RCH=CH_2_ (CH_3_O)_2_CH_2_	GS, KS, AS, A1B, A1C, A2B, A2C, G1A, G1B, G1C, G2A, G2B, G2C, K1B, K1C, K2B, U1C, U2B, U2C
5.32	CH_2_Cl_2_	GS, G1A, G1B
6.2–6.7	RCH=CH_2_	A1B, A2B, G1A, G1B, G2A, K1B, K2B, K2C, U1C, U2B, U2C
5.5–7.5	Phenolic compounds	KSB, A1B, A2B, A2C, G1B, G2A, G2B, G2C, K2B, U1C
7–7.2	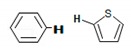	A1B, A2B, G1A, G1B, G2B, G2C, KS, K1B, K1C, K2B, K2C, U1C, U2B, U2C
7.4–7.9	Furan Naphthalene Methenamine Imidazole	KS, A1B, A2B, G1A, G1B, G1C, G2B, K1B, K2B. U2C
8.1–8.7	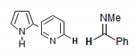	G2A

Grahamstown samples: G1A (wastewater, autumn), G2A (treated effluent, autumn), G1B (wastewater, winter), G2B (treated effluent, winter), G1C (wastewater, spring), G2C (treated effluent, spring), GS (sludge). King Williams Town samples: K1B (wastewater, winter), K2B (treated effluent, winter), K1C (wastewater, spring), K2C (treated effluent, spring), KS (sludge). Alice samples: A1B (wastewater, winter), A2B (treated effluent, winter), A1C (wastewater, spring), A2C (treated effluent, spring), AS (sludge). Uitenhage samples: U1B (treated effluent, winter), U1C (treated effluent, spring).
